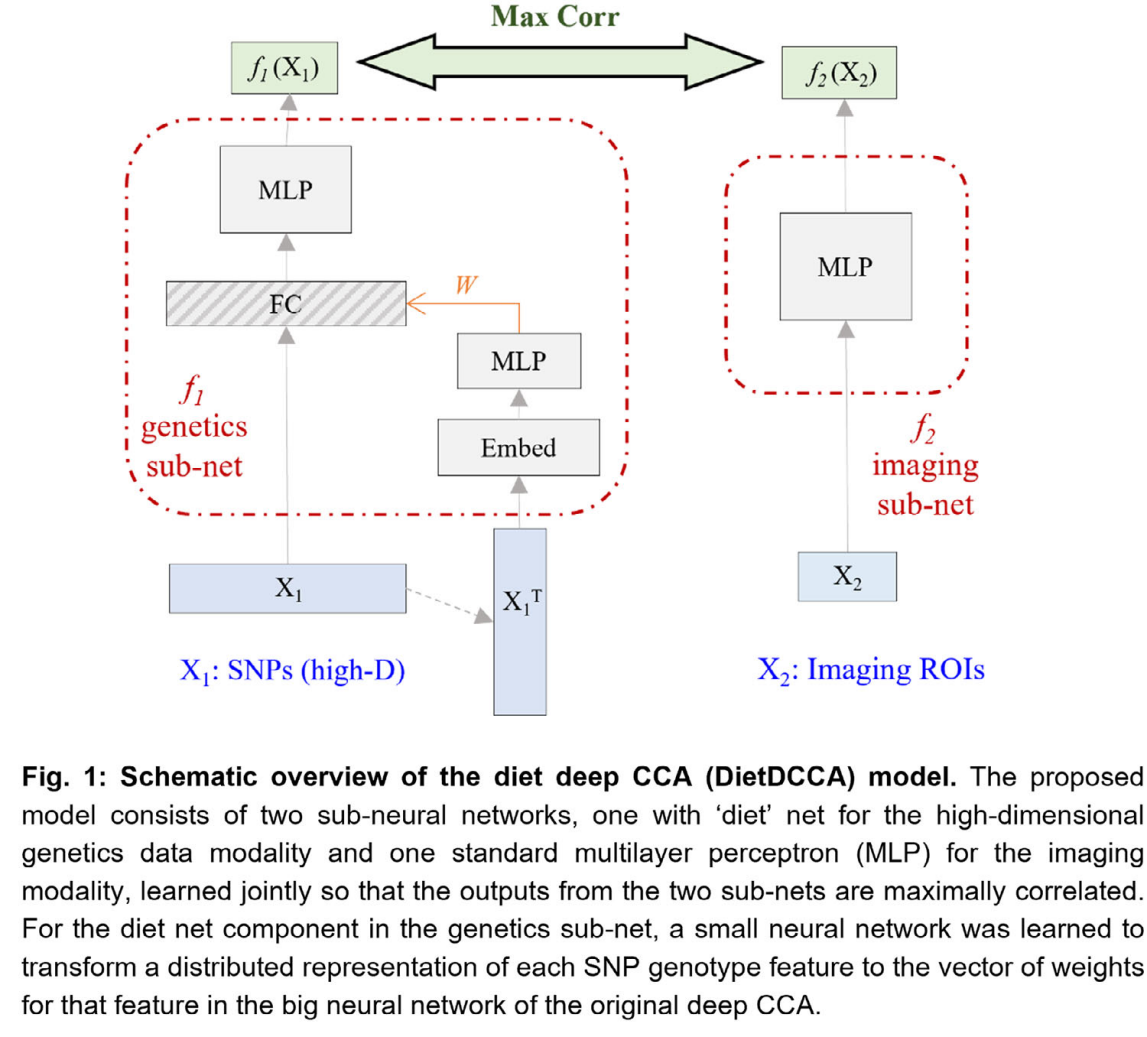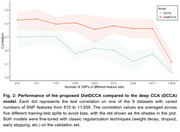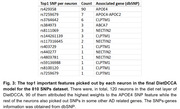# ‘Diet’ deep canonical correlation analysis for high‐dimensional genetics study of brain imaging phenotypes in Alzheimer’s disease

**DOI:** 10.1002/alz.092330

**Published:** 2025-01-09

**Authors:** Shu Yang, Austin Wang, Jingxuan Bao, Shizhuo Mu, Yanbo Feng, Zixuan Wen, Jae Young Baik, Junhao Wen, Bojian Hou, Rongguang Wang, Heng Huang, Andrew J. Saykin, Paul M. Thompson, Christos Davatzikos, Li Shen, for the ADNI and AI4AD

**Affiliations:** ^1^ University of Pennsylvania, Philadelphia, PA USA; ^2^ University of Maryland, College Park, MD USA; ^3^ Indiana University, Indianapolis, IN USA; ^4^ University of Southern California, Los Angeles, CA USA

## Abstract

**Background:**

Understanding the relationship between genetic variations and brain imaging phenotypes is an important issue in Alzheimer's disease (AD) research. As an alternative to GWAS univariate analyses, canonical correlation analysis (CCA) and its deep learning extension (DCCA) are widely used to identify associations between multiple genetic variants such as SNPs and multiple imaging traits such as brain ROIs from PET/MRI. However, with the recent availability of numerous genetic variants from genotyping and whole genome sequencing data for AD, these approaches often suffer from severe overfitting when dealing with ‘fat’ genetics data, e.g. large numbers of SNPs with much smaller numbers of samples.

**Methods:**

Here, we propose to tackle the challenge by integrating an efficient model parameterization approach from Mila’s Diet Network architecture into DCCA to handle high dimensional SNP data in AD imaging‐genetics study (Figure 1). The new method, DietDCCA, was applied to nine datasets derived from 955 subjects in the ADNI data. Each dataset contains 68 FreeSurfer cortical ROIs from the florbetapir (AV45) PET imaging and varied numbers of SNPs from 810 to 11,938 based on different significance thresholds derived from previous studies.

**Results:**

Firstly, we compared our DietDCCA with DCCA on each of the nine datasets to demonstrate the improvement on test correlations (Figure 2). DietDCCA outperformed DCCA by a large margin on all datasets even when the SNPs are as many as ∼10k. Next, we sought to verify if the detected correlations were contributed by meaningful SNPs and extracted the SNP feature that has the largest weight in each neuron of the diet net layer (Figure 3). DietDCCA successfully selected the APOE4 SNP (rs429358) for most cases and also picked out SNPs in other genes (ABCA7, APOC2, CLPTM1, NECTIN2) that were previously reported to associate with AD.

**Conclusions:**

We introduced a novel method, DietDCCA, to handle high‐dimensional SNP features in AD imaging‐genetics study. The initial investigation of DietDCCA on the ADNI data showed promises in detecting correlation signals with AV45 ROIs from biologically meaningful SNPs. The study supplies a novel and effective tool to study the genetic basis of AD imaging phenotypes for future analyses.